# Staphylococcus pseudintermedius induces pyroptosis of canine corneal epithelial cells by activating the ROS–NLRP3 signalling pathway

**DOI:** 10.1080/21505594.2024.2333271

**Published:** 2024-03-22

**Authors:** Zhihao Wang, Long Guo, Changning Yuan, Chengcheng Zhu, Jun Li, Haoran Zhong, Peng Mao, Jianji Li, Luying Cui, Junsheng Dong, Kangjun Liu, Xia Meng, Guoqiang Zhu, Heng Wang

**Affiliations:** a College of Veterinary Medicine, Yangzhou University, Jiangsu Co-innovation Center for Prevention and Control of Important Animal Infectious Diseases and Zoonoses, Yangzhou, Jiangsu, China; b International Research Laboratory of Prevention and Control of Important Animal Infectious Diseases and Zoonotic Diseases of Jiangsu Higher Education Institutions, Yangzhou University, Yangzhou, China; c Joint International Research Laboratory of Agriculture and Agri-product Safety of the Ministry of Education, Yangzhou, Jiangsu, China; d National Reference Laboratory for Animal Schistosomiasis, Shanghai Veterinary Research Institute, Chinese Academy of Agricultural Sciences, Shanghai, China

**Keywords:** *Staphylococcus pseudintermedius*, canine corneal epithelial cells, intracellular infection, pyroptosis, NLRP3 inflammasome, reactive oxygen species

## Abstract

*Staphylococcus pseudintermedius* (*S. pseudintermedius*) is a common pathogen that causes canine corneal ulcers. However, the pathogenesis remained unclear. In this study, it has been demonstrated that *S. pseudintermedius* invaded canine corneal epithelial cells (CCECs) intracellularly, mediating oxidative damage and pyroptosis by promoting the accumulation of intracellular reactive oxygen species (ROS) and activating the NLRP3 inflammasome. The canine corneal stroma was infected with *S. pseudintermedius* to establish the canine corneal ulcer model in vivo. The intracellular infectious model in CCECs was established in vitro to explore the mechanism of the ROS – NLRP3 signalling pathway during the *S. pseudintermedius* infection by adding NAC or MCC950. Results showed that the expression of NLRP3 and gasdermin D (GSDMD) proteins increased significantly in the infected corneas (*p <* 0.01). The intracellular infection of *S. pseudintermedius* was confirmed by transmission electron microscopy and immunofluorescent 3D imaging. Flow cytometry analysis revealed that ROS and pyroptosis rates increased in the experimental group in contrast to the control group (*p* < 0.01). Furthermore, NAC or MCC950 inhibited activation of the ROS – NLRP3 signalling pathway and pyroptosis rate significantly, by suppressing pro-IL-1β, cleaved-IL-1β, pro-caspase-1, cleaved-caspase-1, NLRP3, GSDMD, GSDMD-N, and HMGB1 proteins. Thus, the research confirmed that oxidative damage and pyroptosis were involved in the process of CCECs infected with *S. pseudintermedius* intracellularly by the ROS – NLRP3 signalling pathway. The results enrich the understanding of the mechanisms of canine corneal ulcers and facilitate the development of new medicines and prevention measures.

## Introduction

Bacterial corneal ulcers have often been diagnosed in animals, and *Staphylococcus pseudintermedius* (*S. pseudintermedius*) is a common aetiological pathogen [1–3]. *S. pseudintermedius* belongs to the *S. intermedius* group [4], as an infectious opportunistic pathogen, associated with several animal disorders, including keratoconjunctivitis, otitis externa, and pyoderma [5–8]. Moreover, some *S. pseudintermedius* strains have been proven resistant to quinolone medicines and could survive intracellularly, which was responsible for treatment difficulties [9,10]. Therefore, it is imperative to develop the pathogenic mechanisms of *S. pseudintermedius* to prevent and treat the diseases.

Pyroptosis, a type of programmed cell death mediated by the gasdermin protein families (GSDMs), is involved with inflammatory factors and associated with intracellular infections [11]. It has been reported that fibronectin-binding proteins SpsD and SpsL contributed to the invasion of *S. pseudintermedius* to canine progenitor epidermal keratinocytes [12]. In our previous research, when the canine corneal epithelial cells (CCECs) were infected with *S. pseudintermedius*, the NLR family pyrin domain-containing 3 (NLRP3) inflammasome was activated and mediated the inflammatory response [13]. Upon activation by NLRP3 inflammasome, Caspase-1 can cleave Gasdermin D (GSDMD) to form GSDMD-N, which bounds to phosphatidylinositol phosphates and phosphatidylserine presented in the inner leaflet of cell membrane [14,15], and induces the pyroptosis during the intracellular infection by *S. aureus* [16]. What mechanisms were involved in the CCECs intracellular infection of *S. pseudintermedius*, and what are the relationships among intracellular infection, NLRP3 inflammasome, and pyroptosis? These aspects have not been proven and should be studied in this experiment.

Oxidative stress is defined as a state in which there is a disequilibrium between the production and accumulation of ROS, primarily caused by damage to the mitochondria [17], which has also been associated with intracellular bacterial infection [18]. Accumulation of ROS could trigger activation of the NLRP3 inflammasome [19]. While the activation of the NLRP3 inflammasome could be depressed with N-acetyl-L-cysteine (NAC) or P22phox [20]. ROS production is necessary for NLRP3 inflammasome activation. However, it is not clear whether the activation of NLRP3 inflammasome is related to the accumulation of intracellular ROS in CCECs infected with *S. pseudintermedius* or not.

In this experiment, we aimed to determine whether pyroptosis is involved in the intracellular infection of CCECs. What mechanisms are involved in the interaction between pyroptosis, ROS accumulation, and NLRP3 inflammasome in the ROS – NLRP3 signalling pathway? This would help enrich the understanding of the mechanisms of canine corneal ulcers and facilitate the development of new medicines and prevention measures.

## Materials and methods

### Reagents

Fetal bovine serum (FBS) was acquired from Gibco BRL Co. L-glutamine, Hoechst 33,342 (B2261), propidium iodide (PI, P4170), polyvinylidene difluoride (PVDF), N-acetyl-L-cysteine (NAC), and Dulbecco’s modified Eagle’s medium/F12 (DMEM/F12) were obtained from Sigma-Aldrich. Antibodies against β-actin (#4970), NLRP3 (# 15101), and Caspase-1 (#89332) were purchased from Cell Signaling Technology. Lamin B1 (#ab6048), HO-1 (#ab68477), KEAP1 (#ab227828), and NQO-1 (#ab28947) were purchased from Abcam. Antibodies against NRF2 (#AF0639), GSDMD (#AF4012), GSDMD-N (#DF12275), and HMGB1 (#DF077) were purchased from Affinity Biosciences. The antibodies of IL-1 (#abs126104) were obtained from Absin. The assay kit of BCA protein was acquired from Beyotime Biotechnology. The secondary highly cross-adsorbed antibody (#A32723) used in this study was goat anti-rabbit IgG (H + L), which was obtained from Thermo Fisher Scientific. The ChemiSignal Plus ECL (1810212) was acquired from Clinx Science Instruments Co., Ltd.

### S. pseudintermedius *culture and fluorescence labeling*

*S. pseudintermedius* was isolated from the dog with corneal ulcers in the clinic (NCBI accession number SUB11157782), which was cultured with Luria-Bertani (LB) medium, incubated at 37 °C, with 120 rpm. When the bacteria grew during the log phase, the bacteria were collected and washed twice. Then, it was diluted to achieve 10^6^ CFU/mL.

The *S. pseudintermedius* suspension at the log phase (1 mL) was centrifuged for 5 min at 1200 *g* and then washed with PBS three times. According to Bacterial fluorescence labelling instructions (MycoLight Rapid Fluorescence Gram-Positive Bacterial Staining Kit 22,415, AAT Bioquest, American), 100 μL component B was used to resuspend the precipitate, and it was further subjected to incubate for 20 min at 37 °C after adding 1 μL IF647-ConA stock solution. After centrifuging for 5 min at 1200 *g*, the precipitate was resuspended in 100 μL Component B and kept at 4 °C in the dark.

### Animals

Six healthy Beagle dogs (1–1.5 years old, 15–20 kg) were assigned in this study from Beile Experimental Animal Breeding Co., Ltd. (Jiangsu, China). The animal experiments were reviewed and approved by the Animal Care and Ethics Committee of Yang Zhou University (Approval No: 202011003). The study adhered to the guidelines outlined in the guide for the Care and Use of Laboratory Animals: Eighth Edition, as well as the standards set by the American Veterinary Medical Association [21,22]. The canine corneal ulcer model or control group was constructed with *S. pseudintermedius* (infection group) or sterile PBS (control group) administration into the corneal stromal layer [13].

### Slit-lamp microscope examination, histopathological evaluation, and the expression of NLRP3 and GSDMD detection

The canine eyes were examined by slit-lamp microscope before modelling and at 12, 24, 36, and 48 h after modelling, and photographed and recorded. The scoring criteria referred to in previous reports and were modified as appropriate [13], which details are shown in Table 1.

**Table 1.** Standard for evaluation of corneal opacity, oedema, fluorescein sodium staining, and CNV.

Grande	opacity	edema	fluorescein sodium staining	CNV
0	Completely transparent	No oedema	No staining	No neovascularization
1	Injection site slight opacity	Injection site slight oedema	The injection site has a single stain point	Neovascularization in bulbar conjunctiva
2	Injection site dense opacity	Injection site obvious oedema	The injection site has several scattered staining points	Neovascularization reaches the limbus
3	Partially covering pupil	Cornea oedema over 1/2 area	Scattered points of colouration fuse with each other	Neovascularization pass through the limbus
4	Dense opacity, fully covering pupil	Whole cornea oedema	Ulcer is clearly and pigmented	Neovascularization reaches the ulcer

Canine corneas were collected from the infection and control groups at 48 h after modelling for histopathological examination, immunohistochemical detection, and NLRP3 and GSDMD expression analysis. Details were as follows.

Corneal tissues were fixed in 4% paraformaldehyde for 2 days. Then, the tissues were dehydrated, embedded, and sectioned into 4 μm. HE staining was performed or NLRP3 and GSDMD antibodies were incubated for immunohistopathological stain.

The corneas were utilized to obtain total RNA according to the TRNzol Universal regent instruction. The mRNA expression of *NLRP3* and *GSDMD* was detected by real-time quantitative reverse transcription PCR (qRT-PCR) and the sequence of the primers is shown in Table 2.

**Table 2.** The primes for qPCR used in this study.

Gene	Sequences (5’→3’)	Length (bp)	Accession number
NLRP3	**F**: GAGGAGAAGGCATGGGCCATG	195	XM_005623149
	**R**: CCAATAAACCCAACCACTCCTCTTCAA		
GSDMD	**F**: CCTGTCTGGCACTGCTGTTA	256	XM_049092845
	**R**: TAGGGCCCCTCAAAAACTCG		
GAPDH	F: GGGTGATGCTGGTGCTGAGTAT	186	XM_003435649
	R: TTGCTGACAATCTTGAGGGAGTT		

### Establishment of intracellular infection model and related treatments

CCECs were cultured as previous study [23]. The intracellular infection model was established as follows: the CCECs were infected with *S. pseudintermedius* [MOI = 1:1] for 3 h. Then, 50 μg/mL lysozyme was cocultured for 15 min to clear extracellular bacteria. The medium was discarded, and a new medium was added with the concentration of 20 μg/mL lysozyme, which was regarded as the intracellular infection model and designed as 0 h in the following experiments, and this model was utilized in the following experiments. The details were shown in Table 3 and Supplementary Figure S2.

**Table 3.** The treatment method for each group.

Group	Time (h)		NAC (5 mmol/L)	MCC950 (10 μmol/L)
	0	3		
Control	-	-	-	-
M0	+	-	-	-
M3	-	+	-	-
NAC-M3	-	+	+	-
MCC950-M3	-	+	-	+

Note: “Time” means intracellular infection time; “-” means No; “+” means Yes.

To verify whether the pyroptosis was included in the intracellular infection or not, samples were collected at 0 h (M0 group) and 3 h (M3 group), which were compared to the control group (no infection was included). Secondly, when the intracellular infection model was constructed, 5 mmol/L NAC was cocultured with the cells for 3 hours (NAC-M3 group). The samples were collected, including the NAC-M3 group, M0 group, M3 group, and the control group (no infection was included), which were analysed to prove what functions the ROS played during the infection. Using the same method, 10 μmol/L MCC950 was utilized to culture with the cells (MCC950-M3), and the same groups and times were designed and analysed, which aimed to prove the functions of the NLRP3 played during the infection.

### Intracellular infection detected by transmission electron microscope and laser confocal fluorescence microscope

The cells from M3 and control groups were collected and fixed with 1% osmium tetrachloride solution, dehydrated by anhydrous ethanol, and sectioned into 65 nm thick. Finally, 1% uranyl acetate and 0.1% lead citrate were used for staining, which was observed with transmission electron microscopy (TEM, Tecnai G2 F30 S-TWIN, FEI Company, American).

The CCECs were infected with *S. pseudintermedius* labelled with IF647-ConA as above. The cells were fixed with 4% formaldehyde. The cytoskeleton and nucleus were stained with phalloidin (red) and DAPI (blue), respectively, and observed with the laser confocal fluorescence microscope (Leica TCS SP8 STED, Leica Corp. Germany). The Z-axis was adjusted to scan the cells. The results were analysed in 3D mode using the Leica Application Suite X.

### *Morphological observation of pyroptosis in CCECs induced by* S. pseudintermedius

The cells were infected with *S. pseudintermedius* labelled by IF647-ConA for 5 hours and observed on the live cell station (AXIO Observer 7, Zeiss, Germany). The CECCs were stained with Hoechst 33,342 (5 μg/mL, blue) and PI (2 μg/mL, red) and recorded with the fluorescence microscope or not. The characteristic morphology of pyroptosis with large bubbles was observed continuously.

The cells from M3 and control groups were collected and observed with the scanning electron microscope (SEM). The cells were fixed by 2.5% neutral glutaraldehyde for 12 h, then washed with double distilled water four times, dehydrated by gradient-concentration ethanol, dried at a critical value, sprayed with gold, and observed using the scanning electron microscope (Gemini SEM 300, Zeiss, Germany).

The bright field observation was carried out by phase contrast microscope (BX53, Olympus, Japan), with 200×.

### Colocalization of the cellular membrane and the GSDMD-N protein observed with the immunofluorescence stain

CCECs were cultured with *S. pseudintermedius* labelled with IF647-ConA fluorescently (Purple) as above. Then, the cells were fixed with 4% paraformaldehyde and permeabilized, which were stained with the antibody of GSDMD-N protein (green), DIL (red), and DAPI (blue) in different colours, and observed by laser confocal microscope. The colocalization between GSDMD-N and cell membrane and the fluorescence intensity of *S. pseudintermedius* were analysed by Image J (Java 1.8.0, National Institutes of Health, USA).

### Key protein expression of NLRP3–GSDMD signaling pathway detected with Western blotting

The CEECs were lysed with the RIPA lysis buffer kit, and the total proteins were obtained. SDS-polyacrylamide gels were employed for the separation of the proteins, which were transferred onto polyvinylidene difluoride membranes (PVDF). 5% non-fat milk was applied to the block, and the PVDF was cropped based on the size of different proteins, which were incubated with specified primary antibodies, including pro-IL-1β, cleaved-IL-1β, pro-caspase-1, cleaved-caspase-1, NLRP3, GSDMD, GSDMD-N, and HMGB1 proteins. Then, the secondary antibodies were added and visualized with the ChemiSignal Plus ECL. All the grey values of the proteins were subjected to normalization with β-actin protein.

2.10 Cellular damage detection with LDH assay

The supernatant samples were collected and detected with the activity of the lactate dehydrogenase detection kit according to the instructions of the reagent.

### Intracellular and extracellular bacteria count

Intracellular bacterial number was counted as follows: The CCECs were flushed three times, which were collected and counted. Then, the cells were lysed with 10% Triton-X solution for 20 min, 37, and centrifuged for 5 min, 1200 g. The sediment was suspended with PBS, and the cells were counted with the plate count.

When the extracellular bacteria numbers were detected, the CCECs, which were infected intracellularly, were cultured without 20 μg/mL lysozyme. Then, the culture solution was collected, and the bacteria numbers were analysed with the plate count.

### Pyroptosis rate analysis

The pyroptosis rate of the CCECs was analysed with Caspase-1/PI double staining. The cells were harvested and cultured with the fluorescent-labelled inhibitor of caspases (FLICA) at 4 °C for 1 h. Then, the cellular suspension was centrifuged at 4 °C, 300 g for 5 min and the cells were washed three times. The cells were re-suspended with 100 μL binding buffer and stained by PI for 10 min at 37 °C in the dark. Then, it was mixed with 400 μL binding buffer and detected with the flow cytometer (CytoFLEX S, Becton Dickinson, China). The pyroptosis rate was analysed by the percentage of the cell with Caspase-1/PI double staining positive.

### Detection indicators of oxidative damage-related

According to the instruction of the ROS assay kit (S0033S, Beyotime), the CCECs were harvested and incubated with dichlorodihydrofluorescein diacetate (DCFH-DA) at 37 °C for 30 min. The fluorescence intensity was scanned by the flow cytometer.

The CCECs were collected by the scraper and lysed for 15 min with ultrasound. The supernatant was taken to detect the protein concentration (P0011, Beyotime Biotechnology). According to the corresponding instructions, total antioxidant capacity (T-AOC, A015-2-1), malondialdehyde (MDA, A003-4-1) content, the catalase (CAT, A007-1-1), glutathione peroxidase (GSH-PX, A005-1-2) activities, and total superoxide dismutase (T-SOD, A001-3-2) were detected separately.

The nucleoprotein was extracted using the nuclear and cytoplasmic protein extraction kit (P0027, Beyotime) for the detection of NRF2 protein expression in the nucleus by WB, with Lamin B1 as an internal reference. The other key proteins of the NRF2-KEAP1 signalling pathway were also examined as those in 2.9, including KEAP1, HO-1, and NQO-1 proteins.

### Statistical analysis

All experiments were repeated three times or more. The data were analysed by one-way analysis of variance (ANOVA) and Mann-Whitney U Ranku-Rese test (data do not meet the normal distribution) using SPSS (version 20.0; IBM Corp., ARMONK). After analysis, the data were presented in the form of mean ± standard error of the mean and drawn by GraphPad Prism 9 (Version 9.5.1), where *p <* 0.05 revealed that there was a significant variation between the two groups, *p <* 0.01 revealed there was an extremely significant variation between the two groups.

## Results

### *GSDMD mediated canine corneal ulcers during* S. pseudintermedius *infection*

**Figure 1.**
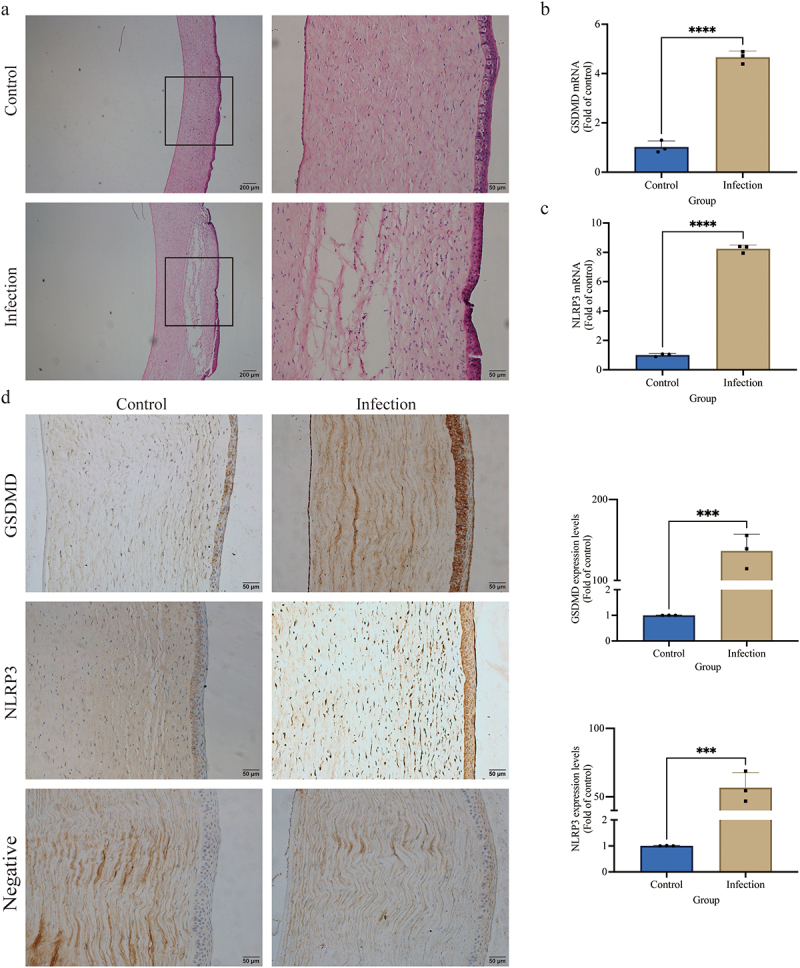
*S. pseudintermedius* can induce the canine corneal ulcer and is related to the GSDMD. (a) The histopathological of canine corneal stained by HE. The mRNA expression of (b) *GSDMD* and (c) *NLRP3* in the corneal tissue of canines was analyzed by the qPCR method (****, *p <*0.0001). (d) The protein expression of GSDMD and NLRP3 in the corneal tissue of canines was analyzed by the immunochemistry method (***, *p <*0.001).

To assess the clinical changes in the established corneal ulcer model, the corneas of the dogs were observed and photographed using the slit-lamp microscope (S Figure 1). Corneal opacity (S Figure 1a), area of oedema (S Figure 1b), CNV (S Figure 1c), and area stained with fluorescein sodium (S Figure 1d) were significantly greater in the infection group compared to the control group at 12, 24, 36, and 48 h (*p <* 0.01). The results of corneal histopathology are shown in Figure 1a. In the infection group, the epithelial layer was absent, the stromal layer was broken, and a large amount of inflammatory cell infiltration was observed. The corneal structure was intact, with only a portion of the epithelium detached from the area of the PBS injection in the control group. Clinical observations and histopathological examinations confirmed that *S. pseudintermedius* could induce corneal ulcers in the canine.

In the infection group, the relative mRNA expression of *NLRP3* and *GSDMD* (Figure 1b, c) was significantly raised compared to the control group (*p <* 0.01). Immunohistochemical staining revealed that the NLRP3 and GSDMD protein expression was elevated significantly (*p <* 0.01) in the infection group in contrast to the control group (Figure 1d). These outcomes show that the GSDMD and NLRP3 protein expressions play an important role in *S. pseudintermedius*-mediated corneal ulceration.

### Staphylococcus pseudintermedius infects CCECs intracellularly and induces pyroptosis

**Figure 2.**
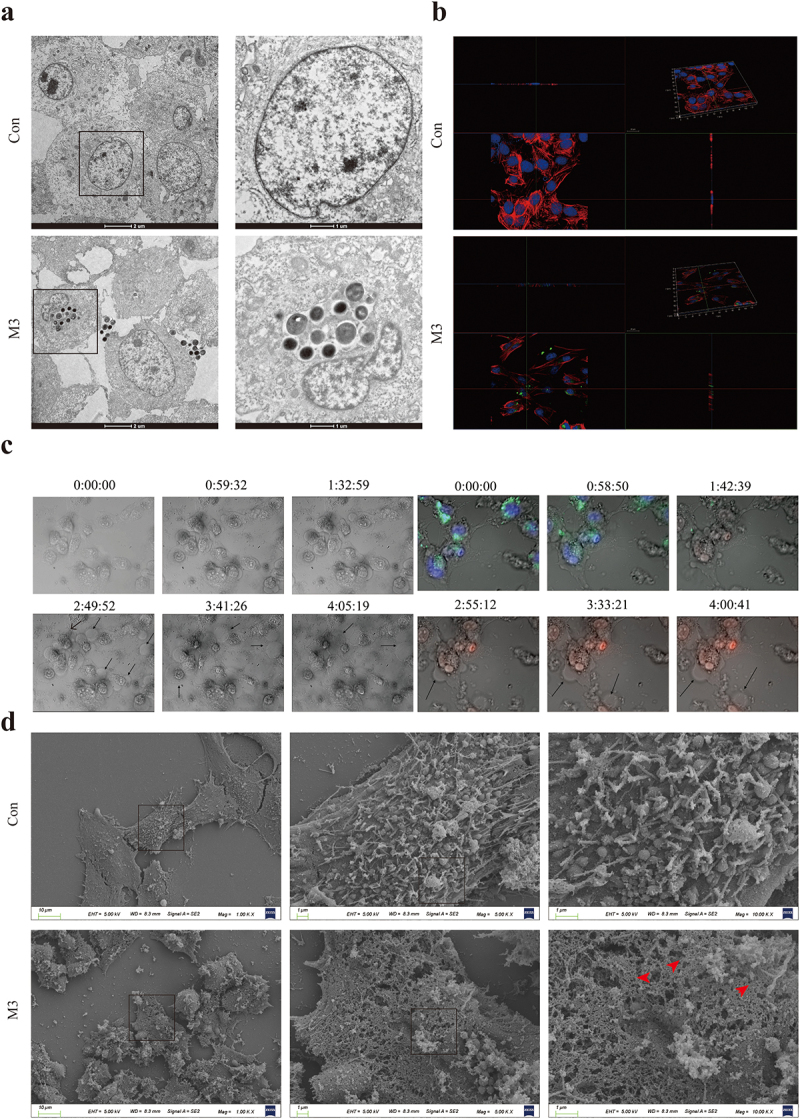
*S. pseudintermedius*intracellularly infected CCEC and caused pyroptosis. (a) The results of transmission electron microscopy, the black spherical bacteria were observed in the group of intracellular infection. (b) Cell nuclei were stained with DAPI (blue), the cytoskeleton was stained with phalloidin (red), and the *S. pseudintermedius* was labeled by IF647-ConA (green). Observing the transverse and longitudinal sections of the cells. (c) The cells were observed by fluorescent microscopy living cell workstation. The black arrow indicated the cell in 2-4 h, beginning with membrane blebbing and producing pyroptotic bodies before plasma membrane rupture. The live cells were labeled with Hoechst 33342 (blue), the dead cells were labeled with PI (red), and the *S. pseudintermedius* was labeled with IF647-ConA (green). (d) Scanning electron microscopy of GSDMD-NT pores on the plasma membrane in *S. pseudintermedius* intracellular infection CCECs. The red arrow indicates the pyroptosis pore.

The results of TEM observation are shown in Figure 2a. In the M3 group, spherical bacteria were observed in the CCECs and at cell junctions, and some bacteria adhered to the cell surfaces, but no bacterial invasion was observed in the control group (Figure 2a). Immunofluorescent 3D imaging detected red, green, and blue fluorescence were observed in the horizontal and vertical sections in the M3 group. This observation indicates that the bacteria were located inside the cells, co-localizing with the nuclei, but no green fluorescence-labelled bacteria have been observed in the control group (Figure 2b). In summary, *S. pseudintermedius* can infect CCECs intracellularly, and the intracellular infection model used in this study was successfully established.

Changes in cell morphology during infection were observed with light-field microscopy and the live cell workstation. The cells in the M3 group showed obvious exocytosis (black arrow). The bacteria escaped from the CCECs, as the infection continued. The cells gradually became swollen, large bubbles were exuded (black arrow), and the cell membrane integrity was compromised (Figure 2c). Ultrastructure examination for SEM showed that the cell membranes in the M3 group were damaged with pores sized 10–21 nm (red arrow), which was characteristic of the cell pyroptosis (Figure 2d).

### Activation of NLRP3–GSDMD signaling pathway during intracellular infection

**Figure 3.**
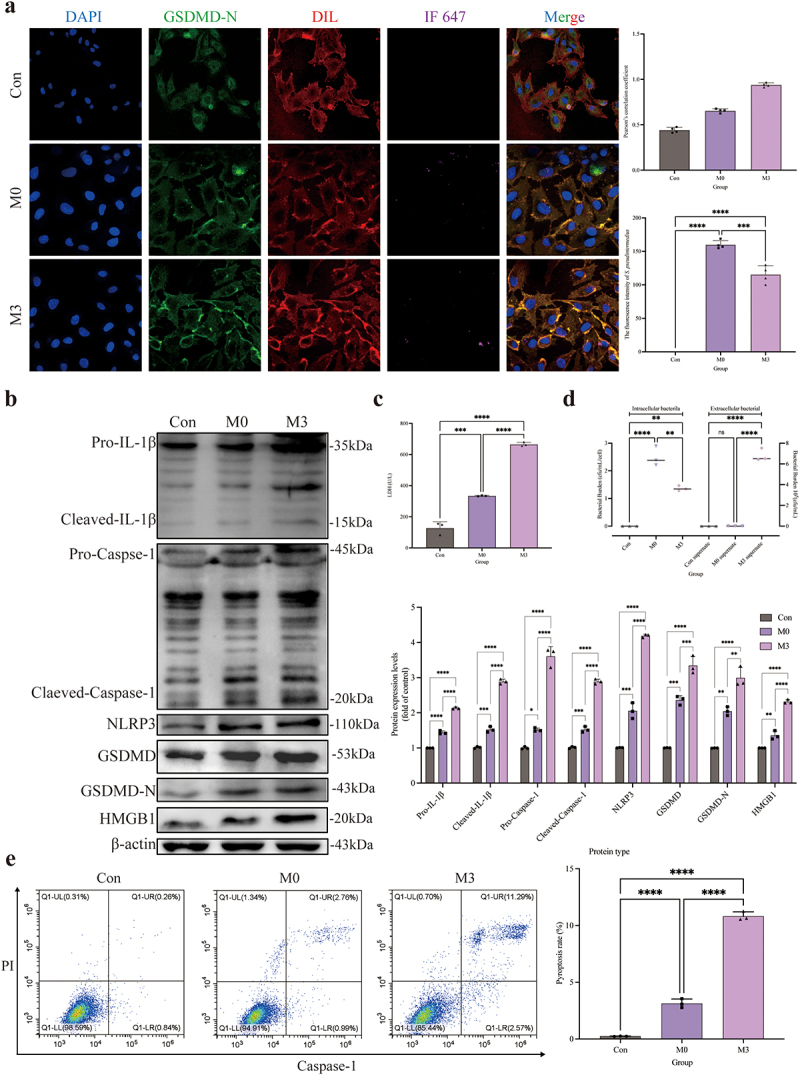
“Con” means control group; “M0” means M0 group; “M3” means M3 group.

The distribution of GSDMD-N was analysed by immunofluorescence assay (Figure 3a). Compared to the control and M0 groups, the GSDMD-N protein accumulated around the cell membrane in the M3 group with a dotted distribution, which is colocalized in the cell membrane by the yellow colour. Pearson’s correlation coefficient was > 0.7, indicating colocalization of GSDMD-N and cell membrane. The fluorescence intensity analysis of *S. pseudintermedius* proved that the intensities of the M3 and M0 groups increased significantly (*p <* 0.01), whereas the intensity of the M3 group was significantly reduced in contrast to the M0 group (*p <* 0.01). The expression of key proteins of the NLRP3-GSDMD signalling pathway was significantly higher in the M0 and M3 groups than those in the control group (*p <* 0.01), including pro-IL-1β, cleaved-IL-1β, pro-caspase-1, cleaved-caspase-1, NLRP3, GSDMD, GSDMD-N, and HMGB1 proteins, which increased significantly in the M3 group than that in the M0 group (*p <* 0.01) (Figure 3b). The results indicated that the NLRP3-GSDMD signalling pathway is involved in the pyroptosis induced by *S. pseudintermedius* in the CCECs.

Intracellular infection of *S. pseudintermedius* activated the NLRP3-GSDMD pathway. (a) Use the *S. pseudintermedius* labelled by IF647-ConA (purple) for intracellular infection [MOI = 1:1] of the CCECs. Cell nuclei were stained with DAPI (blue), the cell membrane was stained with Dil (red), and the GSDMD-N was strained by a specific antibody (green). The area of yellow indicates the co-localization of GSDMD-N and cell membrane. Pearson coefficient score analysis for the co-localization of GSDMD-N and cell membrane. Analysis of the intensity of fluorescence in *S. pseudintermedius*. (b) The cell protein from the Con, M0, and M3 groups was harvested for detecting pro-IL-1β, cleaved-IL-1β, pro-caspase-1, cleaved-caspase-1, NLRP3, GSDMD, GSDMD-N, HMGB1, and β-actin proteins by Western blot. (c) The supernatant during infection was collected for LDH activity detection to reflect the degree of cell damage. (d) The results for the bacterial number from intracellular and extracellular. (e) Pyroptotic cells were measured by flow cytometry using a Caspase-1^+^/PI^+^ pyroptosis detecting kit. (*, *p <*0.05; **, *p <*0.01; ***, *p <*0.001; ****, *p <*0.0001.)

The activity of LDH in the M0 and M3 groups was significantly increased compared to this in the control group (*p <* 0.01), which was significantly higher (*p <* 0.01) in the M3 group than the M0 group (Figure 3c). The results of the bacterial count were shown in Figure 3d. The number of bacteria was significantly higher in the M0 and M3 groups than that in the control group (*p <* 0.01). Nevertheless, the bacterial number in the M3 group decreased significantly compared to the M0 group (*p* < 0.05). Extracellular bacterial detection showed that no bacteria were found in the supernatant of the M0 group, whereas the number of extracellular bacteria was significantly elevated in the M3 group (*p <* 0.01). The results proved the intracellular bacteria escaped. The pyroptosis rate is shown in Figure 3e. The pyroptosis rates were significantly higher in the M0 and M3 groups (*p <* 0.01) than that in the control group, and the rate in the M3 group was significantly higher than that in the M0 group (*p <* 0.01).

### *The oxidative damage induced by intracellular* S. pseudintermedius

**Figure 4.**
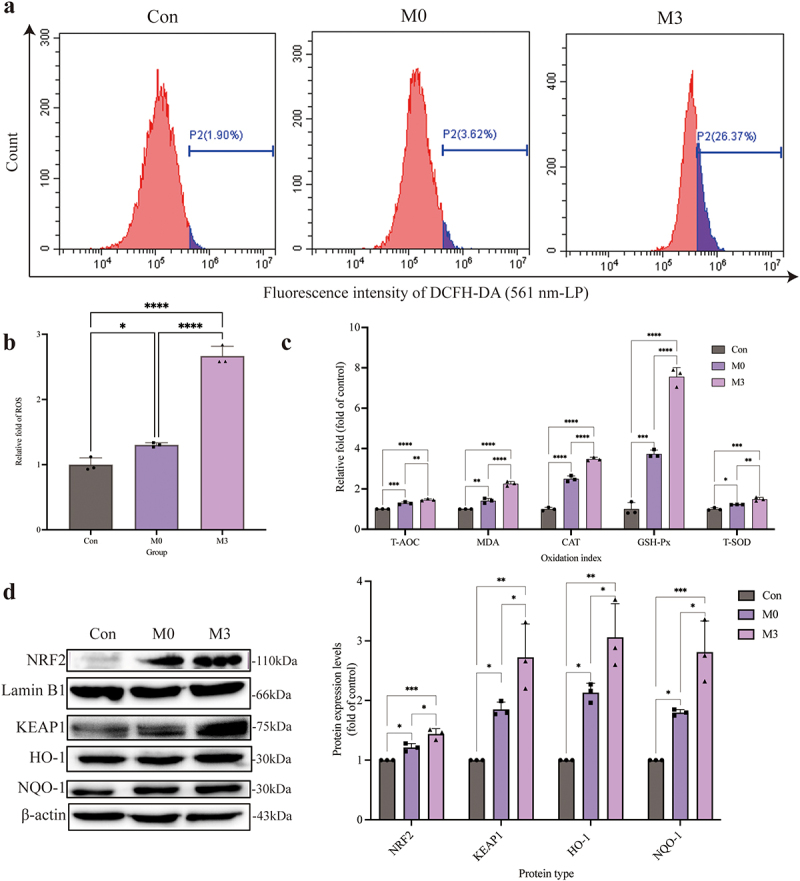
“Con” means control group; “M0” means M0 group; “M3” means M3 group. *S. pseudintermedius* induces oxygen damage during the intracellular infection. (a, b) determined the ROS generation by flow cytometry. (c) The kit detected the expression and activity change of T-AOC, MDA, CAT, GSH-Px, and T-SOD in the cells from the con, M0, and M3 groups. (d) The cell proteins from the con, M0, and M3 groups were harvested for detecting NRF2, lamin B1, KEAP1, HO-1, NQO-1, and β-actin by Western blot. (b-d, *, *p <*0.05; **, *p <*0.01; ***, *p <*0.001; ****, *p <*0.0001.).

Intracellular ROS (Figure 4a, b) and MDA levels (Figure 4c) were significantly increased in the M0 and M3 groups compared to those in the control group (*p* < 0.05 or *p* < 0.01). Meanwhile, the activity of antioxidant enzymes (T-AOC, CAT, GSH-Px, and T-SOD) in the M0 and M3 groups were enhanced, which were higher (*p* < 0.05 or *p* < 0.01) than those in the control group (Figure 4c). The expression of NRF2, KEAP1, HO-1, and NQO-1 proteins, which were the key proteins in the NRF2-KEAP1 signalling pathway, increased significantly in the M0 and M3 groups compared to those in the control group (*p* < 0.05 or *p <* 0.01). In the meantime, which in the M3 group were higher (*p* < 0.05) than those in the M0 group (Figure 4d).

### *ROS influence on the oxidative damage during intracellular infection with* S. pseudintermedius

**Figure 5.**
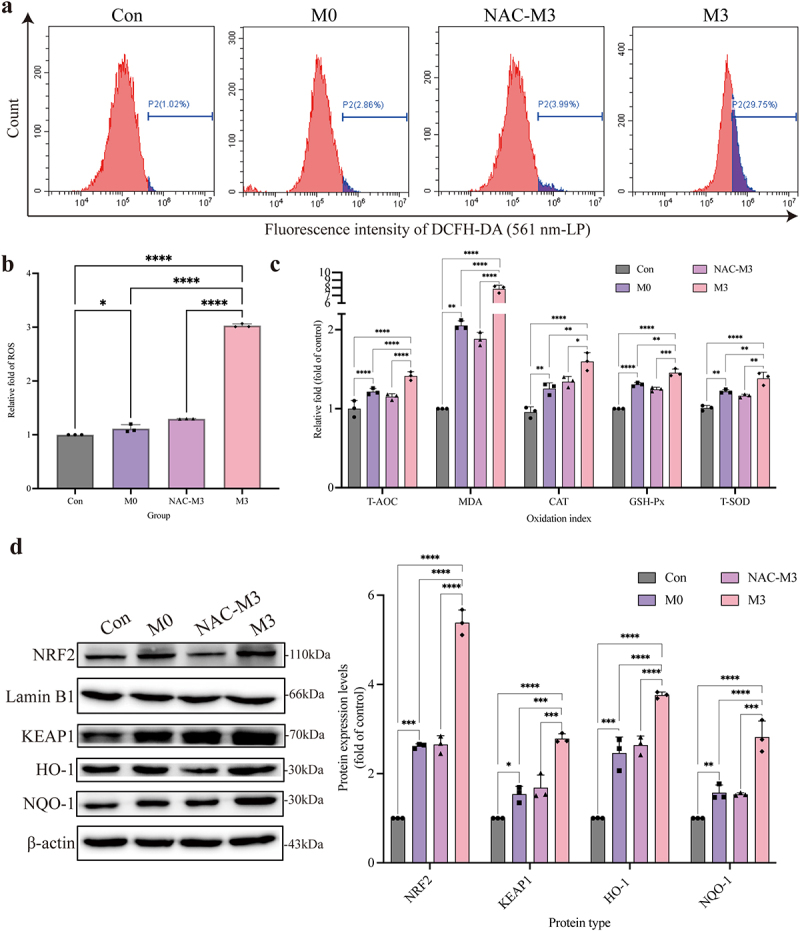
“Con” means control group; “M0” means M0 group; “NAC-M3” means NAC-M3 group; “M3” means M3 group.

Compared to the M3 group, the ROS level in the NAC-M3 group decreased significantly (*p <* 0.01) (Figure 5a, b), which was also observed in the MDA level (*p <* 0.01) (Figure 5c). And the activity of antioxidative enzymes, including T-AOC, CAT, GSH-Px, and T-SOD, in the NAC-M3-group, were depressed in contrast to the M3 group (*p <* 0.01 or *p* < 0.05), which proved that ROS production was induced by *S. pseudintermedius* and clearance of ROS could relieve the reaction of oxidative stress (Figure 5c). The expression of key proteins in the NRF2-KEAP1 signalling pathway in the NAC-M3 group, including NRF2, KEAP1, HO-1, and NQO-1 proteins, decreased extremely (*p <* 0.01) than those in the M3 group (Figure 5d), which verified that the depression of the oxidative stress was related with NRF2-KEAP1 signalling pathway.

Oxidant damage associated with intracellular infection of *S. pseudintermedius*. (a,b) The intracellular ROS levels in CCECs were detected. (c) The activity change of T-AOC, MDA, CAT, GSH-Px, and T-SOD in the Con, M0, NAC-M3, and M3 groups. (d) The cell protein from the Con, M0, NAC-M3, and M3 groups were harvested for detecting NRF2, Lamin B1, KEAP1, HO-1, NQO-1, and β-actin by Western blot. (b-d, *, *p <*0.05; **, *p <*0.01; ***, *p <*NIL0.001; ****, *p <*NIL0.0001.)

### Antioxidant influence on the GSDMD-N translocation and LDH release

**Figure 6.**
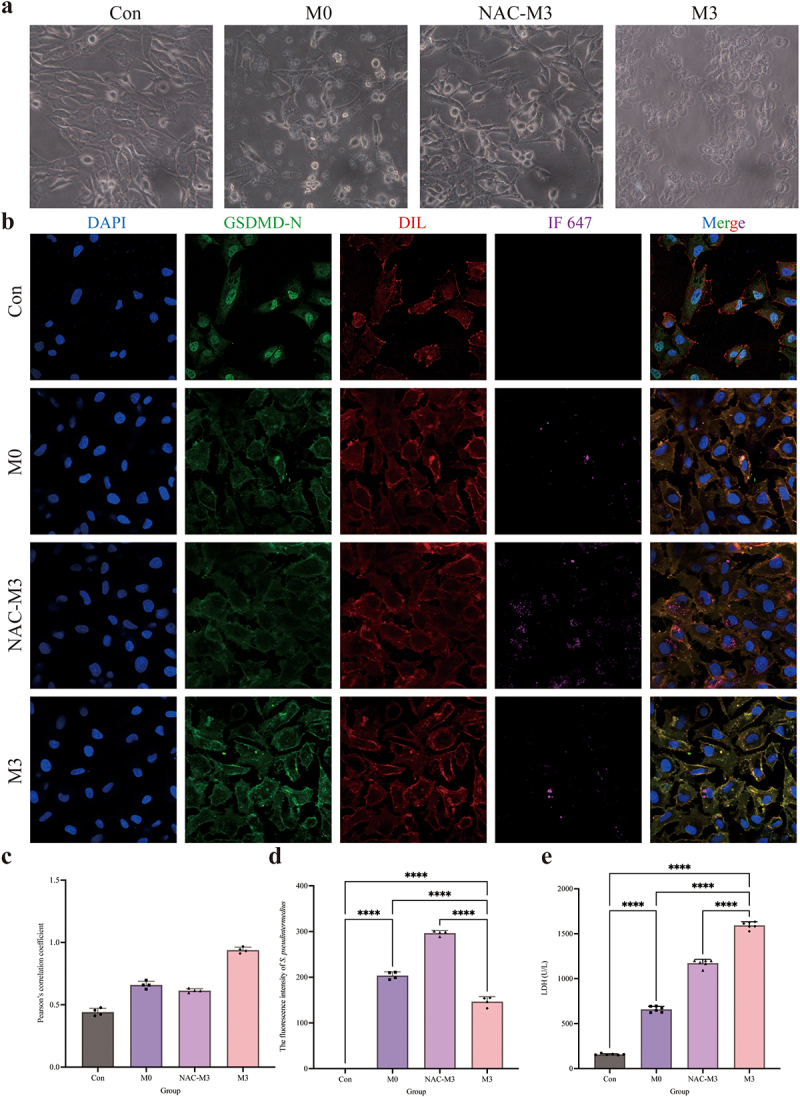
“Con” means control group; “M0” means M0 group; “NAC-M3” means NAC-M3 group; “M3” means M3 group.

In the NAC-M3 group, the extracellular large bubbles decreased obviously under the bright field microscope (Figure 6a), which proved that pyroptosis was related to ROS. When the cells were infected with *S. pseudintermedius* in the M3 group, more GSDMD-N proteins accumulated in the membrane of CCECs, but in the NAC-M3 group, the GSDMD-N translocation was depressed (Figure 6b) with a Pearson coefficient of 0.52 (Figure 6c), which indicated that ROS contributed to the colocation of the GSDMD-N protein and cell membrane. However, the fluorescence intensity of *S. pseudintermedius* in the NAC-M3 group was strengthened significantly (*p <* 0.01), compared to the M3 group (Figure 6d), which proved that the bacteria number increased related to the decrease of ROS production and the depression of the GSDMD-N translocation. The activity of LDH was also reduced significantly (*p <* 0.01) in the NAC-M3 group compared with the M3 group (Figure 6e).

GSDMD-N translocation and LDH release were related to the oxidant damage. (a) Bright-field observation of the Con, M0, NAC-M3, and M3 groups. (b) Cell nuclei were stained with DAPI (blue), the cell membrane was stained with Dil (red), the GSDMD-N was strained by a specific antibody (green), and the *S. pseudintermedius* was labelled by IF647-ConA (purple). The area of yellow indicates the co-localization of GSDMD-N and cell membrane. (c) Pearson coefficient score analysis for the co-localization of GSDMD-N and cell membrane. (d) The analysis of the intensity of fluorescence in *S. pseudintermedius*. (e) Detection of the content of LDH in the Con, M0, NAC-M3, and M3 groups. (c-e, ****, *p <*0.0001.)

### ROS contributed to the pyroptosis and activation of the NLRP3-GSDMD signaling pathway

**Figure 7.**
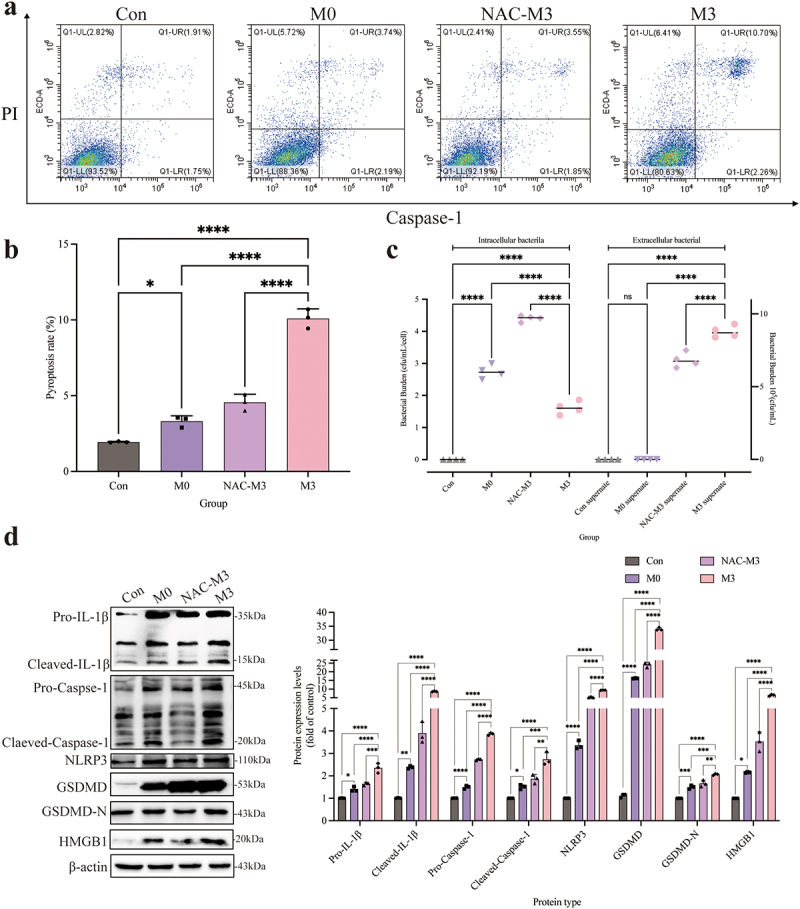
“Con” means control group; “M0” means M0 group; “NAC-M3” means NAC-M3 group; “M3” means M3 group.

To further evaluate the effect of ROS on the pyroptosis, the pyroptosis rate and the number of intracellular and extracellular bacteria were determined. The pyroptosis rate was significantly reduced (*p <* 0.01) in the NAC-M3 group compared with the M3 group (Figure 7a, b). The number of intracellular bacteria was significantly higher (*p <* 0.01) and extracellular bacteria decreased significantly (*p <* 0.01) in the NAC-M3 group, compared to the M3 group (Figure 7c), which demonstrated that pyroptosis was involved in the escape of the intracellular bacteria. In the M3 group, the expression of pro-IL-1β, cleaved-IL-1β, pro-caspase-1, cleaved-caspase-1, NLRP3, GSDMD-N, GSDMD, and HMGB1 proteins were all increased significantly (*p <* 0.01), compared to the NAC-M3 group, which were depressed by the NAC addition. In the meantime, the NLRP3-GSDMD signalling pathway was activated further (*p <* 0.01 or *p <* 0.05) as the time was prolonged in the M0 and M3 groups (Figure 7d). So, the intracellular infection of *S. pseudintermedius* facilitated the activation of the NLRP3-GSDMD signalling pathway and induced pyroptosis, which was relieved with the decrease of ROS.

Pyroptosis rate and the activation of NLRP3-GSMD signalling pathway inhibited by antioxidants. (a, b) Using the Caspase-1^+^/PI^+^ pyroptosis-detecting kit detecting the percentage of pyroptosis in the Con, M0, NAC-M3, and M3 groups. Caspase-1^+^/PI^+^ indicated the pyroptotic cells. (c) Detection of changes in the number of intracellular and extracellular bacteria in the Con, M0, NAC-M3, and M3 groups. (d) The expression changes of key protein pro-IL-1β, cleaved-IL-1β, pro-caspase-1, cleaved-caspase-1, NLRP3, GSDMD, GSDMD-N, HMGB1, and β-actin proteins detected by Western blot at the Con, M0, NAC-M3, and M3 groups. (b-d, *, *p <*0.05; **, *p <*0.01; ***, *p <*0.001; ****, *p <*NIL0.0001.)

### NLRP3 inflammasome activation was conducive to the pyroptosis

**Figure 8.**
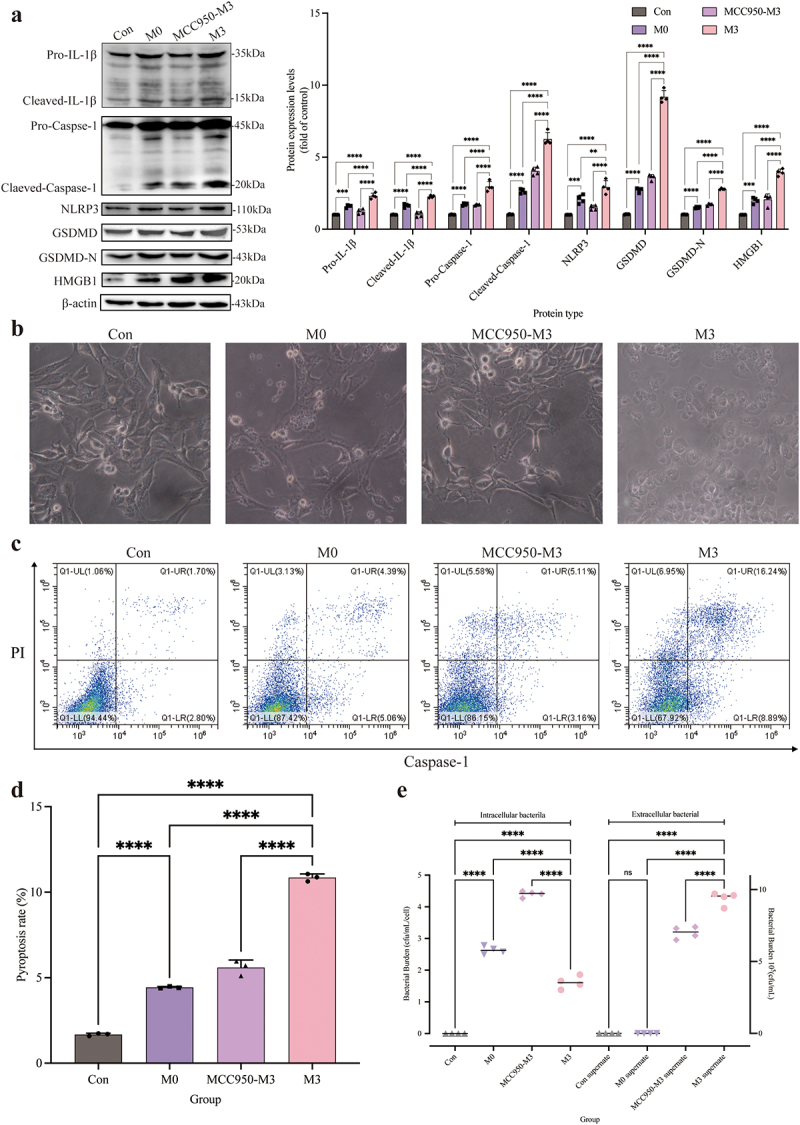
“Con” means control group; “M0” means M0 group; “MCC950-M3” means MCC950-M3 group; “M3” means M3 group.

The pyroptosis of CCECs was induced by *S. pseudintermedius* in the M0 and M3 groups, which was proved by the increase of pro-IL-1β, cleaved-IL-1β, pro-caspase-1, cleaved-caspase-1, NLRP3, GSDMD-N, GSDMD, and HMGB1 proteins significantly (*p <* 0.01), compared to the control group. However, when the NLRP3 inflammasome was inhibited with MCC950 in the MCC950-M3 group, the expression of all the proteins decreased extremely (*p <* 0.01), in contrast to the M3 group (Figure 8a). Which was also proved by the decrease of extracellular large bubbles (Figure 8b) and pyroptosis rate (Figure 8c, d) with inhibition of the NLRP3 inflammasome. And the number of extracellular bacteria in the M3 group increased significantly, which was consistent with a higher pyroptosis rate, compared to the MCC950-M3 group (Figure 8e).

The pyroptosis rate and the NLRP3-GSMD signalling pathway were inhibited by the depression NLRP3 inflammasome. (a) The expression changes of key protein pro-IL-1β, cleaved-IL-1β, pro-caspase-1, cleaved-caspase-1, NLRP3, GSDMD, GSDMD-N, HMGB1, and β-actin proteins detected by Western blot in Con, M0, MCC950-M3, and M3 groups. (b) Bright-field observation of Con, M0, MCC950-M3, and M3 groups. (c, d) Using a Caspase-1^+^/PI^+^ pyroptosis-detecting kit detecting the percentage of pyroptosis in the Con, M0, MCC950-M3, and M3 groups. Caspase-1^+^/PI^+^ indicated the pyroptotic cells. (e) Detection of changes in the number of intracellular and extracellular bacteria in Con, M0, MCC950-M3, and M3 groups. (*, *p <*NIL0.05; **, *p <*NIL0.01; ***, *p <*NIL0.001; ****, *p <*NIL0.0001.)

### Inhibition of the NLRP3 inflammasome reduced GSDMD translocation

**Figure 9.**
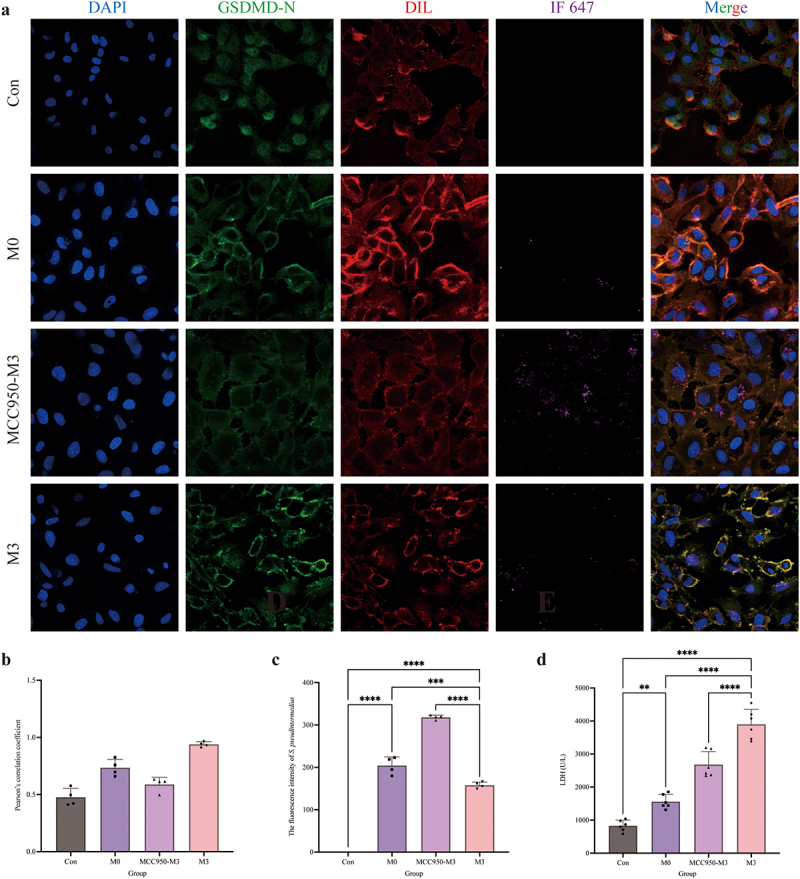
“Con” means control group; “M0” means M0 group; “MCC950-M3” means MCC950-M3 group; “M3” means M3 group.

When CCECs in the M3 group were invaded with *S. pseudintermedius*, colocalization of the GSDMD-N protein and cell membrane was found obviously (Figure 9a). While the colocalization was reduced by the MCC950 coculture in the MCC950-M3 group (Figure 9a) and the Pearson coefficient was 0.53 (Figure 9b) indicated that the colocalization depended on the activation of NLRP3 inflammasome. The fluorescence intensity of *S. pseudintermedius* in the MCC950-M3 group was strengthened significantly (*p <* 0.01), compared to the M3 group (Figure 9c), which proved that the bacteria number increased as blocking the activation of NLRP3 inflammasome and the GSDMD-N translocation. The activity of LDH in the MCC950-M3 group was reduced significantly (*p <* 0.01) compared to the M3 group (Figure 9d). So, pyroptosis was induced by the infection, which facilitated the escape of the bacteria and the cell wound with the LDH release.

GSDMD-N translocation and the release of LDH depend on the NLRP3 inflammasome. (a) Cell nuclei were stained with DAPI (blue), the cell membrane was stained with Dil (red), the GSDMD-N was strained by a specific antibody (green), and the *S. pseudintermedius* was labelled by IF647-ConA (purple). The area of yellow indicates the co-localization of GSDMD-N and cell membrane. (b) Pearson coefficient score analysis for the co-localization of GSDMD-N and cell membrane. (c) The analysis of the intensity of fluorescence in *S. pseudintermedius*. (d) Detection of the content of LDH in the Con, M0, MCC950-M3, and M3 groups. (c, d, **, *p <*NIL0.01; ***, *p <*NIL0.001; ****, *p <*NIL0.0001.)

### Oxidative damage associated with the NLRP3 inflammation

**Figure 10.**
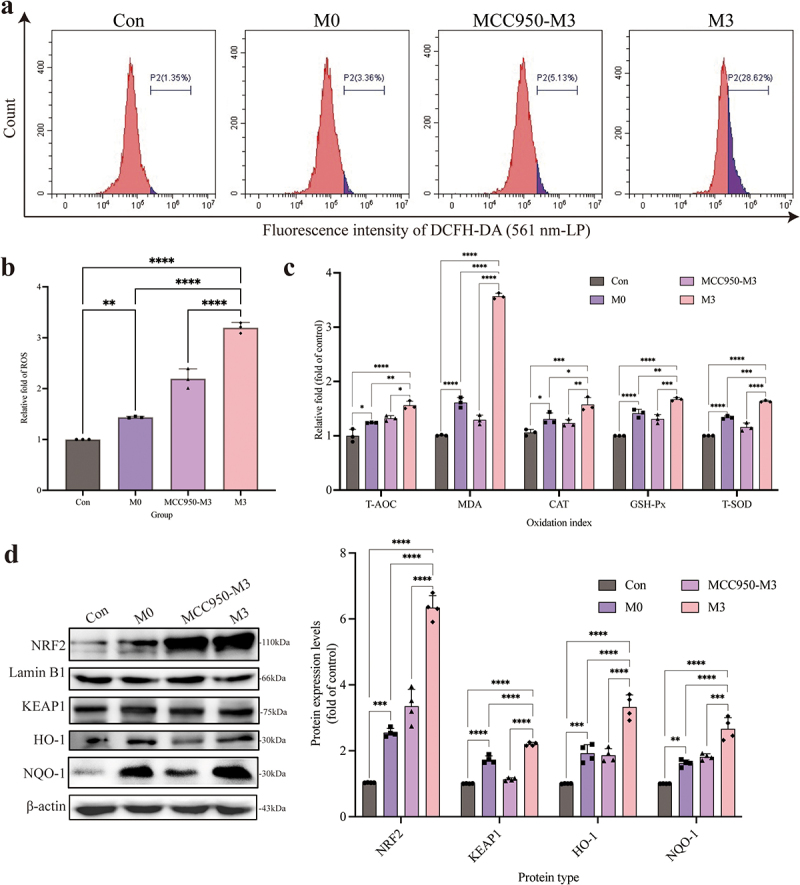
“Con” means control group; “M0” means M0 group; “MCC950-M3” means MCC950-M3 group; “M3” means M3 group.

Inhibition of the NLRP3 inflammasome can decrease the production of the ROS and depress the oxidative stress. Intracellular ROS accumulation in the M3 group increased significantly (*p* < 0.01) with the infection of *S. pseudintermedius*, compared to the MCC950-M3 group (Figure 10a, b). The concentration of MDA and the activity of antioxidant enzymes in the MCC950-M3 group were also decreased obviously (*p* < 0.01 or *p* < 0.05) than those in the M3 group (Figure 10c), which indicated that the oxidative stress was reduced in the MCC950-M3 group. So, the key proteins in the NRF2-KEAP1 signalling pathway were detected. The expression in the MCC950-M3 group of NRF2, KEAP1, NQO1, and HO-1 proteins was significantly inhibited (*p* < 0.01) than those in the M3 group (Figure 10d), which proved that the oxidative stress was relieved with inhibition of the NLRP3 inflammasome and the results were consistent with the depression of pyroptosis mentioned above.

Oxidant damage associated with the NLRP3 inflammasome. (a, b) The intracellular ROS levels in CCECs. (c) The expression and activity change of T-AOC, MDA, CAT, GSH-Px, and T-SOD in the Con, M0, MCC950-M3, and M3 groups. (d) The cell protein from the Con, M0, MCC950-M3, and M3 groups was harvested for detecting NRF2, Lamin B1, KEAP1, HO-1, NQO-1, and β-actin by Western blot. (*, *p <*NIL0.05; **, *p <*NIL0.01; ***, *p <*NIL0.001; ****, *p <*NIL0.0001.)

## Discussion

Corneal ulcers are one of the most common ophthalmic diseases. Causes include trauma, foreign body irritation, systemic immune disease, and eyelid dysfunction. These factors damage the corneal defence barrier, weaken corneal resistance, and/or promote bacterial adhesion to the corneal epithelium, leading to infection. After destroying the inherent corneal barrier, opportunistic pathogens in the conjunctival sac often cause corneal ulcers [24]. Studies have shown that more than a dozen pathogenic bacteria cause bacterial keratitis, predominantly including strains of *S. pseudintermedius*, *S. aureus*, *Pseudomonas aeruginosa*, and *Bacillus cereus*. Gram-positive cocci are the main aetiological agents, followed by Gram-negative bacilli, Gram-positive bacilli, and Gram-negative cocci [1,25,26].

*S. pseudintermedius* is very similar to *S. aureus* in its mode of infection, and intracellular infections by *S. aureus* are predominantly responsible for the diseases it induces, which are consequently difficult to treat [27]. It has been reported that SpsL and SpsD proteins in *S. pseudintermedius* assist in cellular internalization [12], but no intracellular infection model by *S. pseudintermedius* has been established. To fill this research gap, we used TEM to confirm intracellular infection by *S. pseudintermedius*, after which some bacteria divided and proliferated. These findings confirmed the reliability of the intracellular infection model established in the present study. TEM use is limited by the availability of equipment and reagents, lengthy procedures, and high cost. In this experiment, the dye IF647-ConA, which specifically binds to N-acetylglucosamine on the cell wall of Gram-positive bacteria and emits fluorescence at a wavelength of 647 nm, was used to label the bacteria [28]. After CCECs were infected with fluorescent IF647-ConA-labelled *S. pseudintermedius*, the distribution of bacteria in the cells was obvious by immunofluorescence 3D imaging. TEM and immunofluorescence 3D imaging confirmed that *S. pseudintermedius* infected CCECs intracellularly.

NLRP3 inflammation is associated with the occurrence of many diseases and can be activated by a variety of risk signals to mediate the inflammatory response [29–31]. Recent studies have confirmed that ROS and K^+^ outflow are the main signals of NLRP3 inflammation activation. The NLRP3 inflammasome, when fully formed, consists of apoptosis-associated speck-like proteins including a caspase recruitment domain (ASC), NLRP3, and Caspase-1 that has been activated [32]. The specific cleavage of GSDMD protein into GSDMD-N protein by activated Caspase-1 is the main way to mediate pyroptosis [33,34]. For the in vivo experiment, the expression of NLRP3 and GSDMD proteins and mRNAs significantly increased in the corneal tissues of the infected group. In vitro, the key proteins GSDMD, cleaved caspase 1, GSDMD-N, NLRP3, pro-IL1β, HMGB1, pro-caspase 1, and cleaved IL1β of the NLRP3–GSDMD signalling axis were strongly expressed in CCECs infected with *S. pseudintermedius*. These results indicate that the NLRP3 inflammasome and GSDMD-mediated pyroptosis play key roles in corneal tissues infected with *S. pseudintermedius*.

The main manifestation of pyroptosis is the appearance of holes on the cell surface [35]. The study confirmed that these holes were formed by the aggregation of 16 GSDMD-N proteins formed by Caspase-1 cleavage [36]. The gasdermin pore channel is non-selective and H_2_O, Na^+^, K^+^, and Cl^−^ ions can enter the cell, causing cell swelling, cell membrane rupture, and further damage [11]. The gasdermin pore channels evert phosphatidylserine on the cell membrane, allowing PI to enter and stain the nucleus [37]. The pore fluid mediates the efflux of various proteins from the cells, such as cleaved IL1β (4.5 nm) [35], IL18 (5.0 nm), and LDH (9.6 nm) [38], which exacerbates the inflammatory response to the cells. Detection of LDH in the cell culture supernatant reflects damage to the cell membrane. Migration of GSDMD-N to the cell membrane is an indicator of pyroptosis, and the co-localization of GSDMD and the cell membrane can be detected by fluorescence [39]. Pyroptosis is associated with the specific cleavage of gasdermin protein in the caspase family. Gram-positive bacterial infections are mainly caused by the activation of Caspase-1 [40]. Double staining for Caspase-1 and PI effectively determines the extent of pyroptosis [41,42]. In this study, scanning electron microscopy showed that in the M3 group, the cell membranes had 10–25 nm pores, consistent with previous studies, indicating that CCECs had undergone pyroptosis [43]. Our study found that the GSDMD protein is highly expressed in ulcerated corneal tissues. However, changes in the distribution and expression of GSDMD-N protein during intracellular infection of CCECs induced by *S. pseudintermedius* require verification. Immunofluorescence analysis showed that in the M3 group, GSDMD-N translocated from the cytoplasm to the cell membrane and colocalized with the cell membrane. The LDH content in the cell culture supernatant was significantly increased in the M3 group, and the cells were positive for both Caspase-1 and PI. This indicates that CCECs undergo pyroptosis during intracellular infection with *S. pseudintermedius.*

NAC is a thiol-containing antioxidant that increases the free radical capture ability of cell banks and scavenges intracellular ROS [44]. MCC950 is a specific inhibitor of NLRP3 inflammasome, and its mechanism is to inhibit the assembly process of the inflammasome. MCC950 was added during the experiment to verify the role of NLRP3 in validating bodies during infection [45,46]. The addition of NAC or MCC950 during intracellular *S. pseudintermedius* infection of CCECs significantly reduced the accumulation of intracellular ROS and the activity of the NLRP3 inflammasome and inhibited the rupture of the cell membrane. Translocation of GSDMD-N and its co-localization with the cell membrane were also significantly inhibited by treatment with NAC or MCC950. Simultaneously, the fluorescence intensity of intracellular *S. pseudintermedius* was significantly increased. The intracellular and extracellular bacterial counts confirmed that the number of intracellular bacteria increased significantly after the addition of NAC or MCC950, whereas the number of extracellular bacteria decreased. These results indicate that the accumulation of intracellular ROS and NLRP3 inflammasome activation are related to the efflux of intracellular bacteria and the occurrence of pyroptosis. Other studies have reported that the active N-terminus of gasdermin not only pierces the cell membrane surface [11] but also destroys the cell walls of bacteria, thereby killing the invading bacteria [35]. However, it remains to be clarified whether the increase in the number of intracellular bacteria in the cells treated with NAC or MCC950 was achieved by reducing the accumulation of ROS or by activating the NLRP3 inflammasome (thereby inhibiting the killing effect of the GSDMD-N fragment on bacteria).

Flow cytometry showed that in the MCC950 and NAC treatment groups, the expression of caspase 1 decreased significantly, and the caspase 1 and PI double staining indicated the reduction of pyroptosis rate. At the same time, the LDH content in the cell supernatant and the key proteins expression of the NLRP3–GSDMD signal axis, GSDMD-N, NLRP3, HMGB1, pro-caspase 1, cleaved caspase-1, pro-IL-1β, and cleaved IL1β, decreased. These results demonstrate that NAC and MCC950 reduce the rate of pyroptosis by inhibiting the expression of caspase 1 and that the accumulation of intracellular ROS and NLRP3 inflammasome activation are key factors in the activation of caspase-1. They also showed that intracellular infection with *S. pseudintermedius* mediates pyroptosis by activating the NLRP3–GSDMD signalling axis.

Pathological conditions such as ischaemia-reperfusion, heat stress, bacterial infection, and viral infection can induce ROS production [47]. A complete ROS production and clearance system exists in cells in which the NRF2–KEAP1 signalling pathway plays a key role [48]. In the current investigation, we found that during intracellular infection of CCECs by *S. pseudintermedius*, intracellular ROS accumulated, and the MDA content of the cells increased. We also detected changes associated with the antioxidant enzyme activity in the cells, which tended to increase. This result is inconsistent with previous reports in which the antioxidant enzyme activity decreased as the period of infection increased [49,50]. Therefore, we examined the changes in the NRF2-KEAP1 signalling pathway and found that the key proteins NRF2, KEAP1, HO-1, and NQO-1 were strongly expressed. These results suggest that the intracellular accumulation of ROS promotes the dissociation of NRF2 and KEAP1, thereby promoting an increase in the related antioxidant enzyme activity [51]. As a common antioxidant, NAC significantly reduced the levels of ROS and MDA, inhibited the NRF2–KEAP1 signalling mechanism activation, and reduced the activity of antioxidant enzymes during *S. pseudintermedius* infection. At the same time, the NLRP3 inflammasome activation was inhibited, the cleaved-caspase-1 expression declined, and the activation of cleaved IL-1β, GSDMD-N, and HMGB1 was inhibited, thus inhibiting pyroptosis. The accumulation of ROS in cells may activate the NLRP3 inflammasome, which, in turn, promotes the caspase 1 activation and mediates pyroptosis [52]. The findings of our study suggest that the build-up of ROS has a crucial role in the initiation of pyroptosis. The addition of MCC950 also decreased the ROS and MDA accumulation in cells during infection, perhaps by inhibiting the NLRP3 inflammasome activation and reducing inflammatory damage to the cells, consistent with the findings of Bowei Ni [53].

## Conclusion

**Figure 11.**
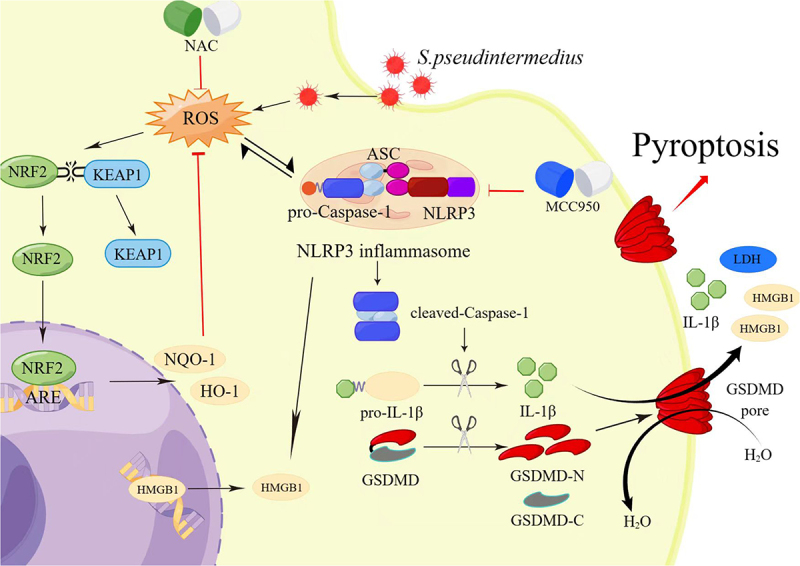
Schematic model: the intracellular infection of CCECs by *S. pseudintermedius* promoted the build-up of intracellular ROS and the formation of NLRP3 inflammasome. Formation of the NLRP3 inflammasome activates caspase-1, which in turn cleaved-IL-1β and GSDMD. After cleavage, the N terminus of GSDMD (GSDMD-N) inserts into the cell membrane, forming pores and inducing pyroptosis (the figure was drawn by Figdraw).

In conclusion, *S. pseudintermedius* can infect CCECs intracellularly, increasing the accumulation of ROS in the cells, which in turn promotes the activation of the NLRP3 inflammasome, which then mediates pyroptosis of the cells (Figure 11).

## Supplementary Material

Supplemental Material

## Data Availability

Data is openly available in a public repository.
