# Supplementary figures and images for: Staphylococcus pseudintermedius induces pyroptosis of canine corneal epithelial cells by activating the ROS–NLRP3 signalling pathway
Source: Virulence. 2024 Mar 22;15(1):2333271. doi: 10.1080/21505594.2024.2333271 (PMC10984133; doi:10.1080/21505594.2024.2333271)

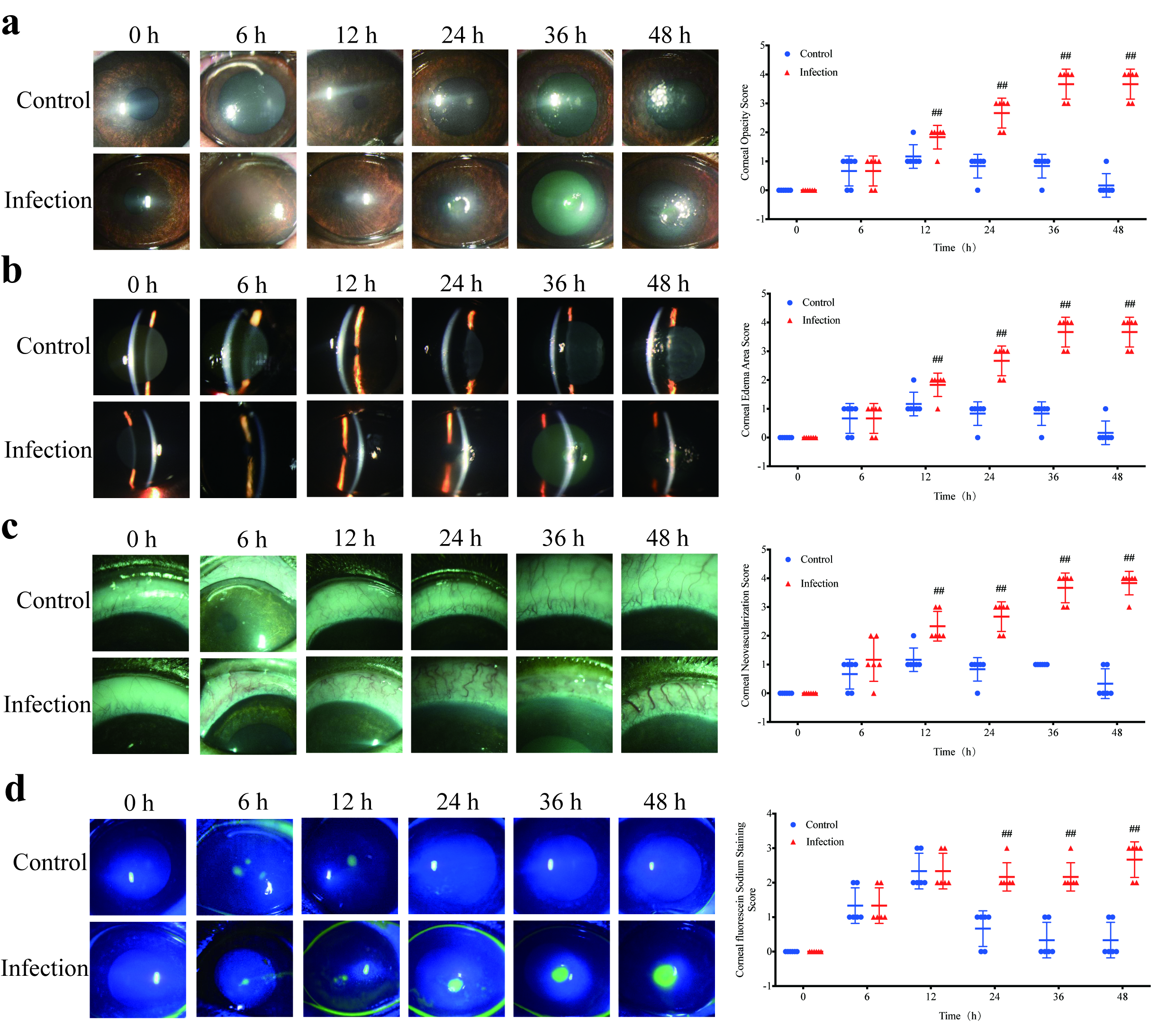

Supplement: Supplemental Material [file KVIR_A_2333271_SM7293.zip › S Figure 1.tif]

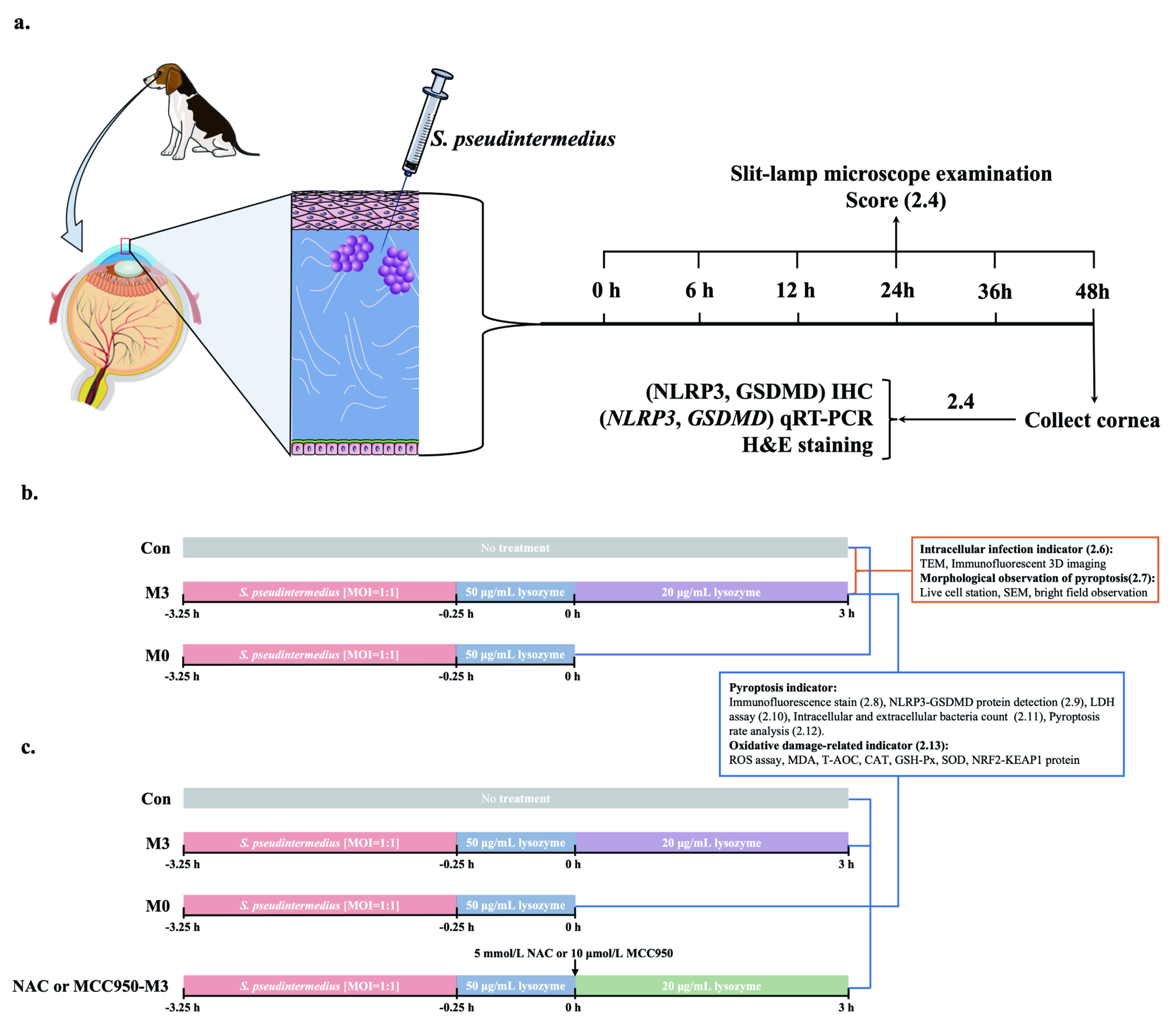

Supplement: Supplemental Material [file KVIR_A_2333271_SM7293.zip › S Figure 2.tif]

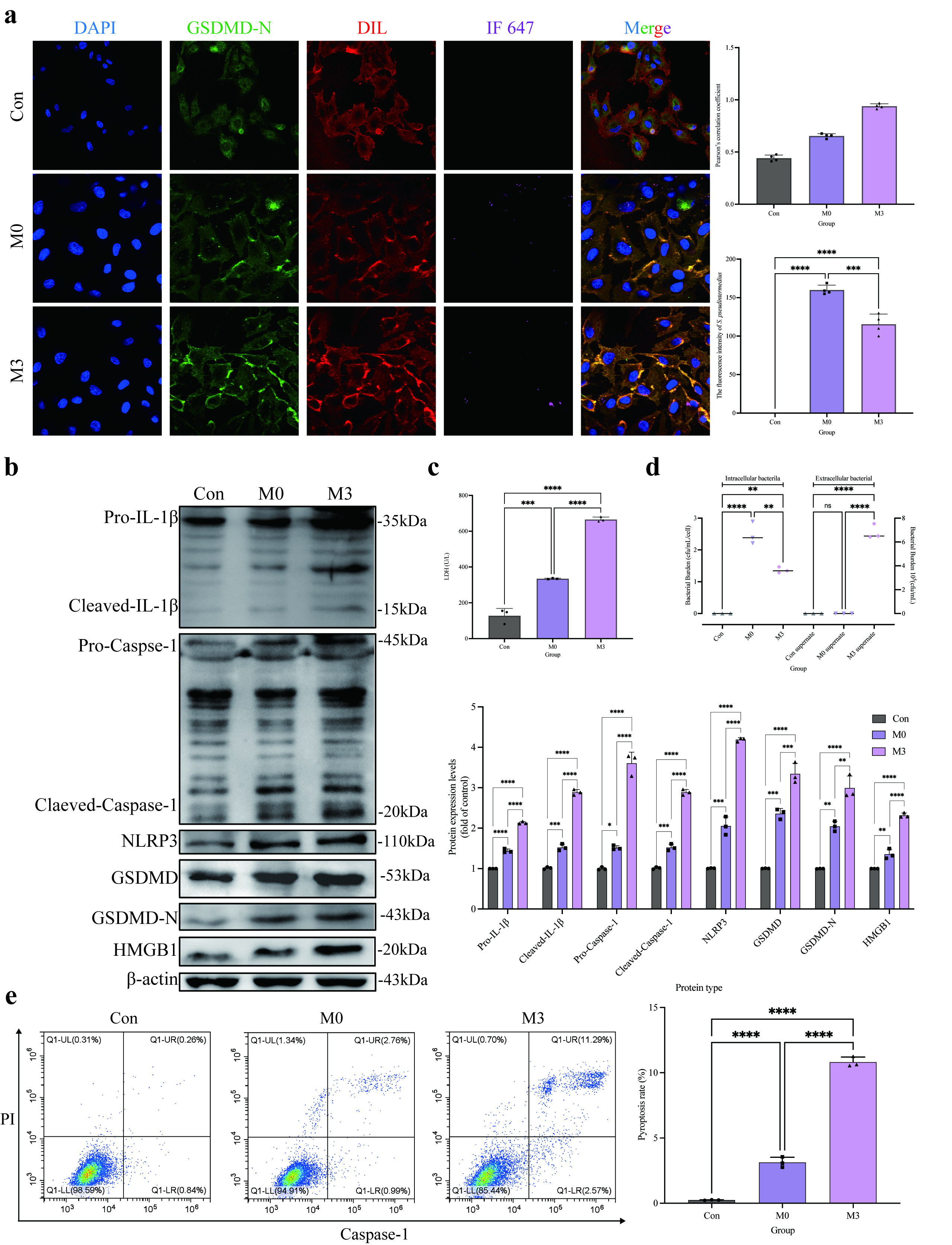

Supplement: Supplemental Material [file KVIR_A_2333271_SM7293.zip › S Figure 3.tif]

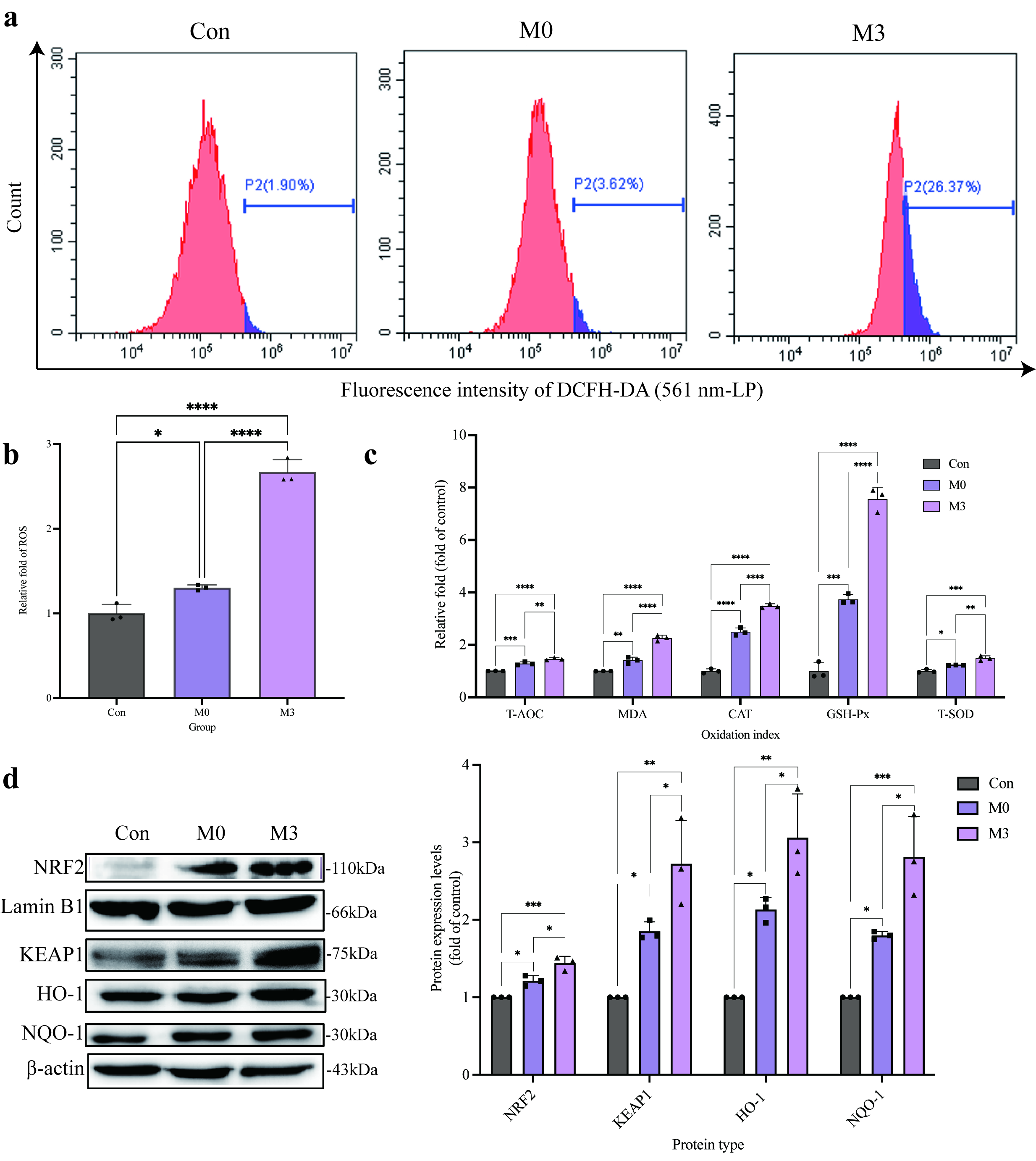

Supplement: Supplemental Material [file KVIR_A_2333271_SM7293.zip › S Figure 4.tif]

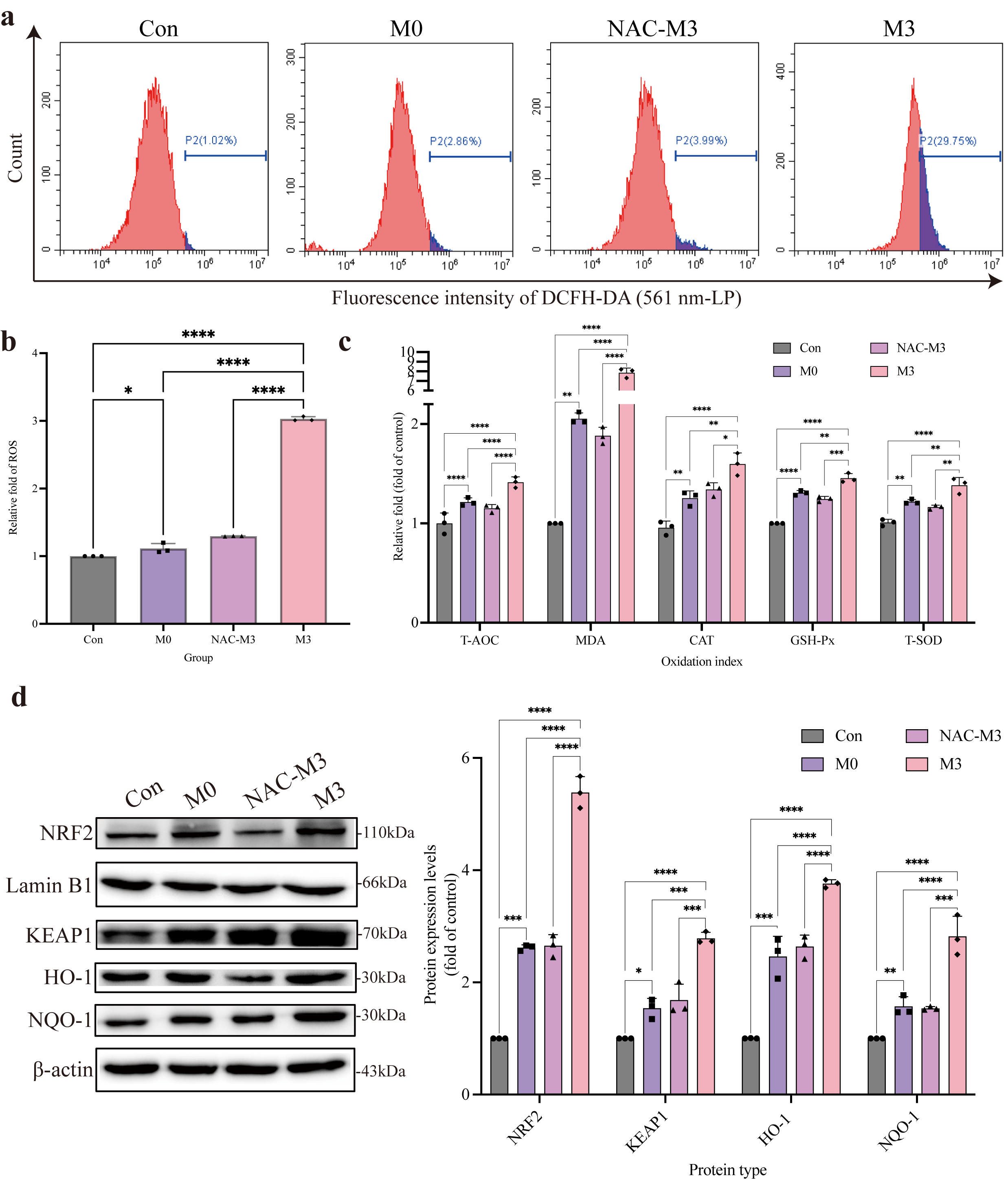

Supplement: Supplemental Material [file KVIR_A_2333271_SM7293.zip › S Figure 5.tif]
